# Beyond the Activity-Based Anorexia Model: Reinforcing Values of Exercise and Feeding Examined in Stressed Adolescent Male and Female Mice

**DOI:** 10.3389/fphar.2019.00587

**Published:** 2019-05-17

**Authors:** Imane Hurel, Bastien Redon, Amandine Scocard, Meryl Malezieux, Giovanni Marsicano, Francis Chaouloff

**Affiliations:** ^1^Endocannabinoids and NeuroAdaptation, NeuroCentre INSERM U1215, Bordeaux, France; ^2^University of Bordeaux, Bordeaux, France

**Keywords:** restrictive anorexia nervosa, post-weaning isolation rearing, wheel-running, palatable food, food anticipatory activity, operant conditioning, motivation, reward choice

## Abstract

Anorexia nervosa (AN), mostly observed in female adolescents, is the most fatal mental illness. Its core is a motivational imbalance between exercise and feeding in favor of the former. The most privileged animal model of AN is the “activity-based anorexia” (ABA) model wherein partly starved rodents housed with running wheels exercise at the expense of feeding. However, the ABA model bears face and construct validity limits, including its inability to specifically assess running motivation and feeding motivation. As infant/adolescent trauma is a precipitating factor in AN, this study first analyzed post-weaning isolation rearing (PWIR) impacts on body weights and wheel-running performances in female mice exposed to an ABA protocol. Next, we studied through operant conditioning protocols i) whether food restriction affects in a sex-dependent manner running motivation before ii) investigating how PWIR and sex affect running and feeding drives under *ad libitum* fed conditions and food restriction. Besides amplifying ABA-elicited body weight reductions, PWIR stimulated wheel-running activities in anticipation of feeding in female mice, suggesting increased running motivation. To confirm this hypothesis, we used a cued-reward motivated instrumental task wherein wheel-running was conditioned by prior nose poke responses. It was first observed that food restriction increased running motivation in male, but not female, mice. When fed grouped and PWIR mice were tested for their running and palatable feeding drives, all mice, excepted PWIR males, displayed increased nose poke responses for running over feeding. This was true when rewards were proposed alone or within a concurrent test. The increased preference for running over feeding in fed females did not extend to running performances (time, distance) during each rewarded sequence, confirming that motivation for, and performance during, running are independent entities. With food restriction, mice displayed a sex-independent increase in their preference for feeding over running in both group-housed and PWIR conditions. This study shows that the ABA model does not specifically capture running and feeding drives, i.e. components known to be affected in AN.

## Introduction

Anorexia nervosa (AN), which mainly affects older adolescent and young adult females (with a sex ratio of 8 for 1 male), is a psychiatric disorder where self-starvation and hence dramatic underweight is a core symptom (Kaye et al., 2009; Zipfel et al., 2015). As opposed to a general belief, it is unlikely that socio/cultural influences play a major, if not unique, role as AN was already reported centuries ago (Casper, 2006). Its lifetime prevalence in high-income countries is ∼1–4% (Smink et al., 2012; Zipfel et al., 2015; Keski-Rahkonen and Mustelin, 2016), with a constant increase in that percentage over recent years (Smink et al., 2012). However, AN, whether restrictive or associated with purgative behavior, is not solely accounted for by a decreased drive for feeding. In many cases, especially in restrictive anorexia, this decrease is associated, and often preceded by, motor restlessness and/or an increased drive for another reward, i.e. exercise, mostly running (Brewerton et al., 1995; Davis, 1997; Klein et al., 2004; Meyer et al., 2011; Casper, 2018). Reinforcing the hypothesis that increased exercise is at the core of AN are the reports that i) exercise dependence might be one cause of altered eating behavior (Cook and Hausenblas, 2008), ii) remitted AN patients still display craving for exercise (Shroff et al., 2006), and that iii) the latter amplifies the anhedonic profile of these patients (Davis and Woodside, 2002). It is this imbalance between energetic supply and energy consumption rates that provides AN with severe and often lethal consequences. Although AN etiology is ill-defined (Clarke et al., 2012), family and twin studies have indicated that AN patients are at risk to transmit the disease to their progeny (Bulik et al., 2007; Zipfel et al., 2015). However, the identification of AN genetic defects is rendered complex as this disease is not accounted for by one single gene (Bulik et al., 2007). Besides familial causes, environmental risk factors have also been delineated. Among these, perinatal (e.g., prematurity, imbalanced maternal control) and/or childhood trauma have been underlined (Leung et al., 2000; Romans et al., 2001; Canetti et al., 2008; Pike et al., 2008; Zipfel et al., 2015). This might explain why patients suffering AN display comorbidity with mood disorders, including major depression, anxiety, and obsessive-compulsive disorders (Kaye et al., 2009; Zipfel et al., 2015). With respect to childhood trauma, physical abuse, sexual abuse and parental neglect are the more documented forms of social stress that might, in combination with genetic or other environmental factors, precipitate AN (Yackobovitch-Gavan et al., 2009; Jaite et al., 2012; Racine and Wildes, 2015). The negative impact of childhood trauma is further illustrated by the report that post-traumatic stress disorder and AN might actually co-occur (Reyes-Rodríguez et al., 2011). The observation that early traumatic events provide a long-term psychoneuroendocrine vulnerability to future stressors in laboratory rodents (Lupien et al., 2009; McCormick et al., 2016) provides support for an etiological role of early trauma in AN.

To date, the model considered to be the most pertinent for AN—although it is unlikely that a single model recapitulates such a complex pathology—is the so-called “activity-based anorexia (ABA)” paradigm (Boakes, 2007; Scheurink et al., 2010; Kim, 2012; Mequinion et al., 2015). Thus, rodents housed with a running wheel and placed under a severe restricted feeding regimen (i.e., a single time- or quantity-limited access to food per day) display a progressive increase in running activity at the expense of feeding. Such an increase is mainly accounted for by high wheel-running activity prior to food delivery (namely food anticipatory activity, FAA). After several days, body weight loss is so pronounced (up to 30%) that death might occur, especially in rats (Routtenberg and Kuznesof, 1967). Beyond methodological limits that might question the causal relationship between food scarcity and physical hyperactivity (Dwyer and Boakes, 1997; Rowland et al., 2018), the validity of the ABA paradigm as an animal model of AN might be discussed with regard to the construct, face, and predictive validity criteria thought to define any model of human (psycho)pathology (see Willner, 1984). This is especially true for the construct criterium in which factors thought to be of etiological significance in AN pathology should thus logically bear consequences in the ABA model. In keeping with the data reported above, genetics, sex (female *vs*. males), age (adolescence *vs.* adulthood), and early traumatic stimuli are expected to have significant impacts in the ABA model. As opposed to genetic studies, which provide thorough evidence that the consequences of the exposure to the ABA model depend on the rat/mouse line tested therein (Pjetri et al., 2012; Klenotich et al., 2012), studies aimed at investigating the respective impacts of sex and age in this model have provided contradictory results (Mequinion et al., 2015; Rowland et al., 2018). As opposed to genetics, sex, and age, available data on the impact of early traumatic stimuli in the ABA paradigm are somewhat scarce. Prenatal stress (Boersma et al., 2016; Schroeder et al., 2018), early weaning (Glavin and Pare, 1985) or postnatal separation (Carrera et al., 2009; Hancock and Grant, 2009) have shown diverse effects, including when considering the animal sex. Although these studies addressed the consequences of prenatal and perinatal stress manipulations that might bear translational value with respect to AN, the question of the impact of stress during childhood and early adolescence should be considered. As mentioned above, physical and/or sexual insults during these periods have long-lasting psychological consequences, especially in females where such stressors increase the propensity to develop affective disorders (Bale and Epperson, 2015). Of major relevance to the present focus, childhood and early adolescence trauma can be modeled in rodents through the so-called post-weaning isolation rearing (PWIR) stress paradigm. Actually, rodents housed individually immediately after weaning (21 days in rodents), and thus deprived of social contacts, display long-lasting emotional disturbances (e.g., anxiety, cognitive rigidity, aggression, proneness to drug self-administration; Fone and Porkess, 2008; Walker et al., 2019) that might be translationally relevant to AN in humans.

Herein, we first studied the consequences of PWIR on wheel-running performances in an ABA paradigm wherein food-restricted female mice were provided a limited amount of food at the onset of the dark cycle. Because the core of AN is an imbalance between the respective motivation drives for exercise and feeding (Klein et al., 2004; Casper, 2006; Keating, 2010; Keating et al., 2012), we next asked whether the impact of PWIR in the ABA found its origin at the motivation level. To do so, we shifted to an operant conditioning procedure wherein mice needed to nose-poke to unbrake a running wheel (Muguruza et al., 2019). This procedure allowed us to examine how i) food restriction and ii) PWIR respectively affected running motivation. As AN involves decreased motivation for feeding, we finally asked the question of i) the impact of PWIR on motivation for palatable food before ii) examining the respective drives for wheel-running and palatable feeding under *ad libitum* and food restricted conditions when these rewards were made concurrent (Muguruza et al., 2019).

## Materials and Methods

### Animals

All protocols, which complied with the French (Décret 2013–118) and European (2010/63/EU) rules on animal experimentation, were approved by the local Ethic Committee (Comité d’Ethique 50) with agreement numbers DIR13111, 13649, 33-063-69 (F.C.) and A33-063-098 (animal facilities) provided under authority of the Préfecture de Gironde and the Ministry of Agriculture. Accordingly, the 3R-rules were followed, including through the use of the minimal number of animals per series of experiments that was required to reach conclusions. In addition, in keeping with the procedures used in this study (see the methodological outline), and which could have long-lasting consequences, all animals were only used once and sacrificed thereafter.

This study mainly used 3-week-old male and female C57BL/6N mice (Elevage Janvier, Le Genest Saint Isle, France). Upon arrival in our animal facilities, these mice were housed either singly (PWIR) or in three to four (group-housed). This study also involved 8-week-old male and female C57BL/6N mice, all individually housed (to avoid inter-individual aggression). All animals were housed in a thermoregulated room (21–22°C) placed under a partly inverted 12-h light/12-h dark cycle with lights off at 2:00 PM (free wheel-running experiments) or at 10:00 AM (operant conditioning experiments). Excepted for experiments involving restriction feeding regimen (see below), mice were provided with food and water *ad libitum*.

### Methodological Outline

**Figure 1:**
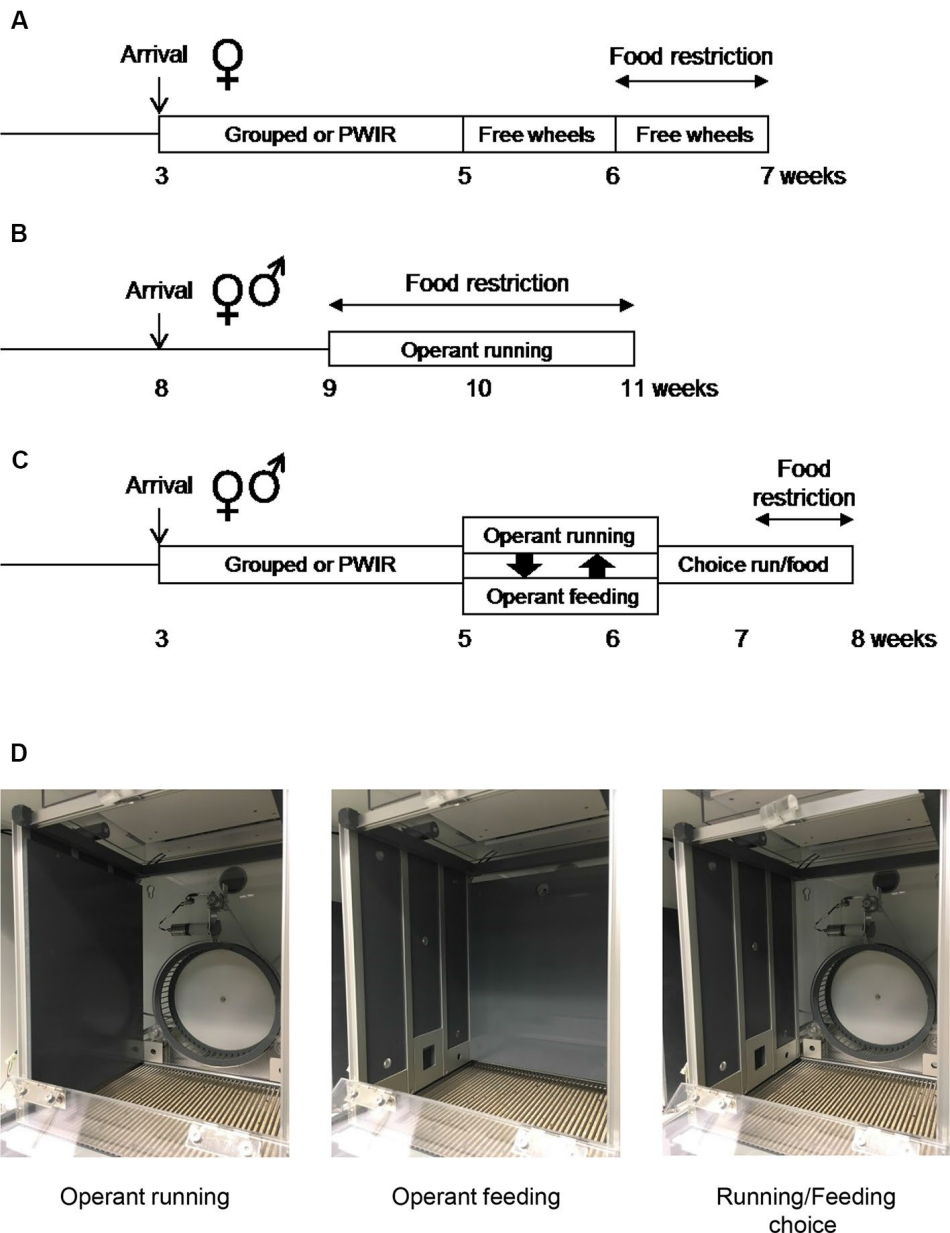
Experimental protocols and operant chamber set-ups. **(A–C)** Protocol schemes for the three series of experiments aimed respectively at investigating the effects of post-weaning isolation rearing (PWIR) on free wheel-running in food-restricted mice **(A)**, of the mouse sex on running motivation under fed and food restriction conditions **(B)**, and of the mouse sex and of PWIR on the choice between running and feeding under fed and food restriction conditions **(C)**. **(D)** Operant chamber set-up for the study of running motivation alone (left), palatable feeding motivation alone (center), and the preference between running and feeding in a concurrent choice design (right).

A first series of experiments involved group-housed and PWIR female mice provided with wheels in their home cages under *ad libitum* fed conditions before being food-restricted (ABA protocol; **Figure 1A**). A second series of experiments involved individually-housed fed and food-restricted male and female mice, these mice being conditioned to nose poke for access to running wheels located in operant chambers (wheel-running motivation; **Figure 1B**). A third series of experiments used group-housed and PWIR male and female mice which were conditioned to nose poke for access to wheel-running or palatable food, these rewards being first proposed alone before being proposed in competition under fed and, then, food-restricted conditions (**Figure 1C**).

### Activity-Based Anorexia Protocol

At the age of 5 weeks, group-housed mice and mice singly-housed after weaning were singly placed in cages housing a running wheel (25-cm diameter, Intellibio, Seichamps, France). Following a 7-day period of habituation to their new environment during which food intakes, body weights and daily running activity were monitored, mice were then placed under a food-restriction procedure for another 7-day period (**Figure 1A**). This restriction procedure consisted in the daily placement of a limited amount of food (50% of the mean daily intake measured during the preceding week) in each cage, this amount being provided (after having checked for the absence of food crumbs) at the onset of the dark part of the light/dark cycle. Body weights were monitored daily while wheel-running performances were recorded on an hourly basis.

### Operant Conditioning Set-Up

Motivation for wheel-running and/or food intake was studied in 12 individual operant chambers (28 cm long × 26 cm wide × 38 cm high) located in a room adjacent to the animal housing room, as previously described (Muguruza et al., 2019). These chambers were placed inside wooden casings (60 cm long × 62 cm wide × 49 cm high) that were ventilated to guarantee air circulation and to provide background noise (Imetronic, Pessac, France). For operant running experiments, lateral walls were made of gray Perspex while the rear wall had a central hollow for mounting the 20-cm-diameter wheel, the release trigger of which was connected to a circuit enabling the wheel to be locked or unlocked (by means of a brake-pad) in accordance with predefined experimental conditions (**Figure 1D**, operant running configuration). A cue-light placed above the wheel indicated the wheel unlocking. The wheel was flanked by two small ports (2.5 cm above the chamber grilled floor with cue lights located above) set into the rear wall to allow the animal to “poke” its nose through. For operant feeding, the rear side (running wheel, nose poke ports, cue-lights) was covered by gray Perspex whereas the left panel of the chamber housed in its center a recessed pellet tray surrounded by two nose poke (nose poke) ports (**Figure 1D**, operant feeding configuration). Cue-lights were placed above the nose poke ports and the feeder to indicate respectively effectiveness of the nose poke and pellet distribution. For reward choice sessions, the above-mentioned Perspex walls were removed to allow conditioned wheel-running or conditioned feeding (**Figure 1D**, running/feeding choice configuration). Nose poke performance could be either “active” (leading to cue-light illumination and wheel unlocking or cue-light illumination and pellet distribution) or “inactive” (having no consequence). Left/right allocation of active/inactive nose poke ports was counterbalanced between animals during experiments. All devices in the operant chambers were linked to a computer which recorded both the number of active/inactive nose poke, the number of running sequences, and the running duration/distance covered during each rewarded sequence (wheel-running configuration), and the number of active/inactive nose pokes, the number of pellets distributed, and the number of entries into the feeder (feeding configuration). Food pellets were 20-mg chocolate-flavored pellets composed of 59.1% glucids, 18.4% proteins, 5.5% lipids, 6.5% minerals and 4.6% fibers (72 cal per 20-mg F05301 BioServ pellet; Plexx, Elst, The Netherlands).

### Operant Conditioning Protocols

All protocols were similar to those already reported (Muguruza et al., 2019). In one series of experiments aimed at assessing the respective influences of the animal sex and of food restriction on wheel-running motivation (see above), operant conditioning procedures involved training under fixed-ratio 1 (FR1) and FR3 schedules of wheel-running reinforcement followed by a progressive ratio (PR) schedule of reinforcement. In a second series of experiments aimed at assessing the respective influences of the animal sex, of PWIR and of food restriction on wheel-running motivation and on feeding motivation in a choice paradigm, operant conditioning procedures first involved training under FR1 and FR3 schedules of wheel-running or palatable food intake reinforcements, each reward being available alone. These training procedures were then followed by a PR schedule of reinforcement for each reward. Mice were then returned to one session of FR3 schedule reinforcement with wheel-running and palatable food intake being reinforced separately. Thereafter, mice were placed under additional FR3 schedules of reinforcement with both rewards being provided in a choice paradigm. The selection of one reward temporarily excluded any possibility to obtain the second reward. In all experiments, food-restricted mice, whether tested for running motivation or for palatable food motivation, were provided their daily chow at least 1 h after their operant session. Daily food provision, which was calculated as to promote a 10% reduction in initial body weights, took into account the amount of food eaten during the preceding test session. The time schedule that we chose, i.e., motivation tests 1–2 h before feeding, thus allowed to examine running and feeding drives at time periods corresponding to those during which FAA was observed in the ABA protocol.

For the first series of experiments (**Figure 1B**), male and female mice singly housed for a week, and aged 9 weeks old, underwent one daily habituation session in the operant chambers for two consecutive days. Mice were placed in the operant chambers with the cue light above the unlocked running wheel remaining illuminated while the two nose poke ports were covered-up by metal pieces. These two 60-min sessions were aimed at habituating the mice to both the operant chamber, the wheel and the cue indicating wheel-unlocking. When learning sessions began (**Figure 1D**, operant running configuration), the wheel locking/unlocking mechanism and the nose poke ports were fully operational. The wheel was unlocked for 60 s (wheel brake release) following nose pokes the mouse executed in its allocated active nose poke port. In the FR1 condition, a single active nose poke was sufficient to simultaneously illuminate the cue-light above the port for 10 s, unlock the running wheel for 60 s and illuminate a light above the wheel. Nose pokes in the other port were counted but were without functional consequence. When the 60-s period had elapsed, the wheel-light extinguished and the brake applied, so that the mouse had to step down from the wheel and execute a further nose poke in order to unlock it again. Nose pokes made in the active port while the wheel was already unlocked, counted as uncorrect responses, were without consequence. Habituation and FR1 sessions were ran once daily and lasted for 60 min. After completing six FR1 sessions, mice moved on to the FR3 condition, i.e., a 60-s wheel-running period was contingent on three consecutive nose pokes in the active port. The day after the last FR3 session mice were tested under a linear PR schedule of reinforcement where i) the number of active nose pokes required to free the running wheel was incremented by three between each rewarded step (three, six, nine … etc: PR3), with ii) a time limit of 15 min between two successive steps.

For the second series of experiments (**Figure 1C**), group-housed mice and PWIR mice were first habituated to the 20-mg food pellets by providing them 3 to 5 pellets/day in their home cages for the 3 days that preceded their first day of exposure to the operant chambers. On this first day of habituation to the chambers, mice were exposed to two consecutive 30-min sessions with the running wheel being unlocked during the first session (**Figure 1D**, operant running configuration) while during the second session, the feeder distributed 17 chocolate pellets (**Figure 1D**, operant feeding configuration). In between, mice were returned for 5 min in their home cages (with drinking water) as to allow operant chamber configuration changes (wheel to food or *vice versa*). During these two sessions, whose reward order was counterbalanced, cue lights above the unlocked running wheel or the pellet tray remained illuminated while nose poke ports were covered-up by metal pieces for each configuration. These habituation periods were followed by a conditioning phasis wherein animals learned the contingency between the introduction of the muzzle into the “active” nose poke port and the access to the related reward. For this purpose, nose poke holes were not masked anymore as to allow the mouse to “poke” its nose through. As for habituation, two consecutive sessions per day (30 min/session) were performed: one for food (50% of the individuals in each mouse group) and the second for wheel-running (the remining 50% of the individuals in each mouse group), the order between the sessions being daily alterned. To facilitate the learning of the contingency for food (and hence running), mice were first food-restricted (as to display a stable 10% body weight reduction) for the first two to three FR1 sessions, i.e., sessions during which a single nose poke was sufficient to illuminate the cue light above the wheel or the food port for 5 s. Simultaneously the cue light above the wheel was activated for 20 s (indicating the possibility to run) while that above the food magazine was activated for 15 s (indicating the distribution of one food pellet). Although mice consumed their food pellet rapidly, we decided not to shorten the rewarding periods as i) to allow sufficient time for running and ii) to avoid rapid food satiety. Wheel unlocking or pellet distribution was respectively followed by 20- and 15-s time-out periods during which nose poke activity was inefficient. Five sessions of FR1 for each reward were sufficient to ensure that all animals learned and expressed stable performance over days. Then, animals were placed for another 5-day period under a FR3 schedule wherein three consecutive nose pokes in the active port were required to get one reward (i.e., 20-s wheel running or one chocolate pellet). All mice had a minimal discrimination index of 80% between active and inactive nose pokes. On the two consecutive days that followed the last FR3 session, mice were tested under PR 3 schedule of reinforcements where the number of consecutive active nose pokes required to free the running wheel or to trigger the distribution of one pellet was incremented by three between each rewarded step (three, six, nine…). Half of the mice within each mouse group were tested for wheel-running reinforcement on the first day, the second half being tested for food reinforcement, and vice versa on the second day PR session. PR schedules of reinforcement, by allowing an estimation of the maximal number of consecutive nose pokes performed (and hence the last rewarded step that was reached, i.e., the so-called “breakpoint” level), provide an index of the appetitive motivation for each reward.

### Preference for Wheel-Running Over Palatable Food Consumption

The day after the last PR session, mice from the second series of experiments were returned to FR3 schedules of wheel and food reinforcement as to indicate to the mice that the rewards were again available following a fixed number of active nose pokes. Then, animals were placed in a choice condition (**Figure 1D**, running/feeding choice configuration) with either wheel unlocking or food distribution being accessible under an FR3 schedule (Muguruza et al., 2019). However, choosing one reward excluded the possibility to obtain simultaneously the second reward. The respective durations of activation of the wheel (20 s) and the feeder (15 s) cue-lights remained as in the preceding sessions. However, to further indicate to the mice that might run during the entire 20-s sequence that the reward choice was mutually exclusive, we added a 5-s period during which a green ceiling light was switched on while none of the nose poke ports was active. Five daily consecutive choice sessions were performed to establish food and wheel preferences, each session being 60-min long. To explore how PWIR affected the impact of food restriction on the preference between wheel-running and feeding (as under ABA conditions; see above), these choice sessions were followed by five choice sessions during which the mice were food-restricted (to extents similar to those measured during the first two to three FR1 sessions; see above).

### Data Analyses and Statistics

Measures of wheel-running performances (ABA experiments) were gathered using the ActiviWheel software (Intellibio, France) while operant running and/or feeding data were obtained using the PolyWheel software (Imetronic, France). To evaluate wheel-running consumption during FR/PR sessions in the operant protocols, we divided the total running duration (or the total distance covered) within each session over the number of rewarded events during that session. Additionally, wheel preference (%) in the choice sessions was quantified by dividing the number of active nose pokes that led access to the wheel by the total number of active nose pokes performed for both rewards (food + wheel). Scores above 50% thus indicates a preference for wheel-running while scores below 50% indicates a preference for food.

All data are shown as means ± standard errors of the mean. Two-group (treatment or genotype) comparisons of the data gathered during the PR sessions were achieved by means of two-tailed Student t-tests. Multiple data comparisons were performed through multiple (two- or three-way) analyses of variance (with/without repeated factor), data being log-transformed to achieve variance homogeneity if needed. *Post hoc* comparisons (Tukey test) were only performed if interactions between main variables were significant. In choice experiments, preference scores were compared to non-preference (50% preference for one reward) by one-tailed Student’s t-tests. All analyses were achieved using the GB-Stat 10.0 software (Dynamic Microsystems, USA).

## Results

### PWIR Female Mice Display Increased Food Anticipatory Wheel-Running Activity

**Figure 2:**
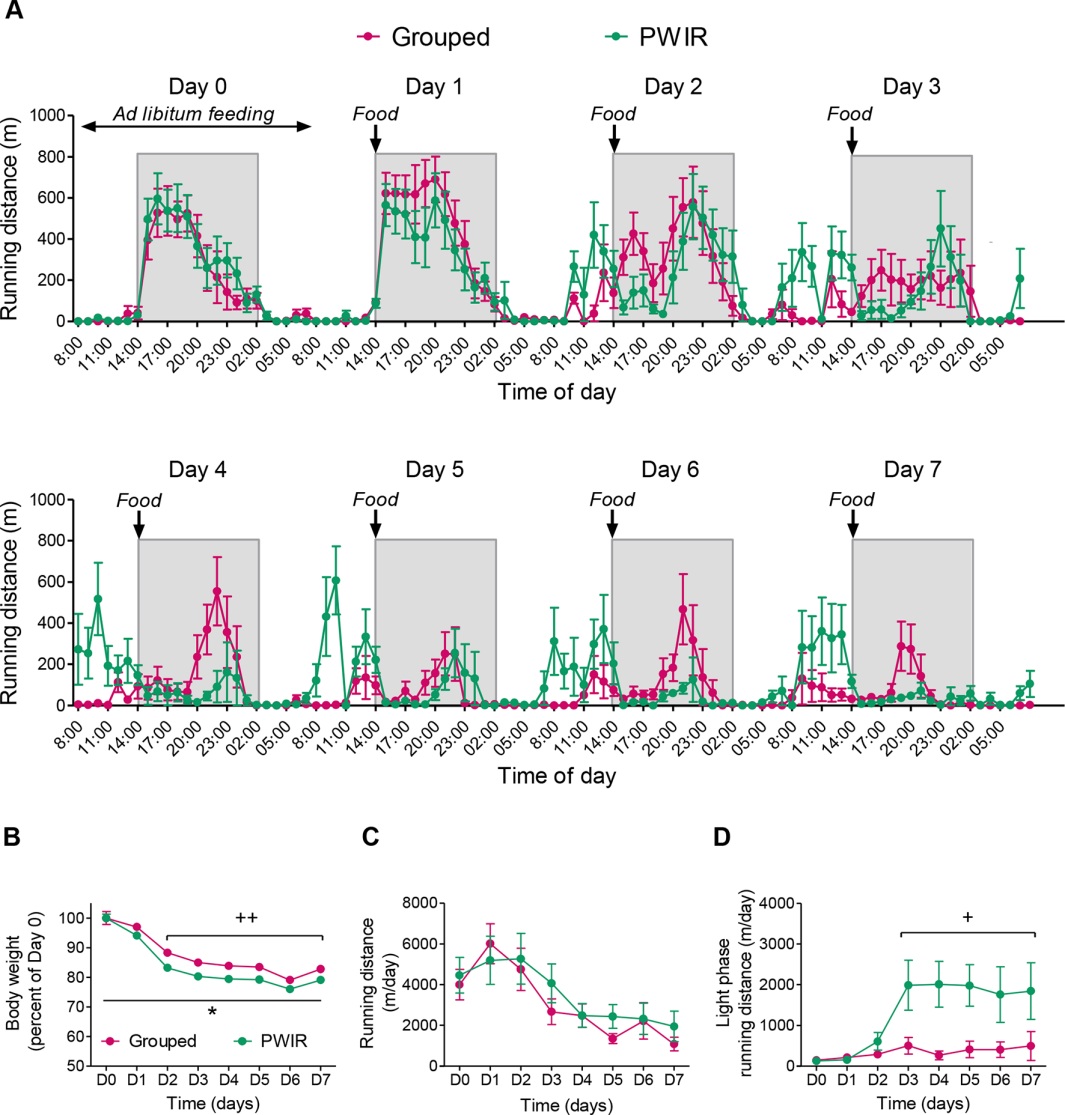
Wheel-running performances of grouped and post-weaning isolation reared (PWIR) female mice submitted to a restricted feeding protocol. **(A)** Hourly wheel-running activities before and during repeated food restriction (days 1–7). A limited amount of food (50% of the food quantity consumed during *ad libitum* feeding conditions) was provided at the daily onset of the dark period of the light/dark cycle. **(B)** Food restriction-elicited body weight reductions in grouped and PWIR mice. **(C,D)** Food restriction effects on daily running distances **(C)** and on daily distances ran during the light part of the light/dark cycle **(D)**. The values are the mean ± standard error of the mean of n = 7–8 mice. * *p* < 0.05 for the impact of PWIR (multiple-way analysis of variance). + *p* < 0.05 and ++ *p* < 0.01 for the difference with D0 (*post hoc* Tukey test following a significant day × mouse group interaction in the multiple-way analyses of variance). D0–D7 refer to day 0–day 7.

Food-restricted grouped and PWIR mice displayed a progressive session-dependent shift of wheel-running activity from the dark part of the nycthemeral cycle to its light part (**Figure 2A**). This shift, which was mainly observed during the hours that preceded food availability (i.e., FAA), concerned to a higher extent the PWIR mice, compared to their grouped counterparts (**Figure 2A**). The overall analysis of wheel-running performances confirmed the latter observation. Thus, food restriction, which decreased body weights in all mice (F_7,91_ = 119.92, *p* < 0.0001), this decrease being larger in PWIR mice (F_1,13_ = 24.84, *p* = 0.0002; **Figure 2B**), inhibited wheel-running activity in both mouse groups (F_7,91_ = 18.26, *p* < 0.0001; **Figure 2C**). However, this overall inhibition was associated with an increased wheel-running activity during the light part of the cycle, hence reflecting increased FAA (F_7,91_ = 7.07, *p* < 0.0001), the amplitude of which was more pronounced in PWIR females, compared to their controls (F_7,91_ = 4.33, *p* = 0.0004 for the time × mouse group interaction; **Figure 2D**).

### Sex-Dependent Effects of Food Restriction on Wheel-Running Motivation

**Figure 3:**
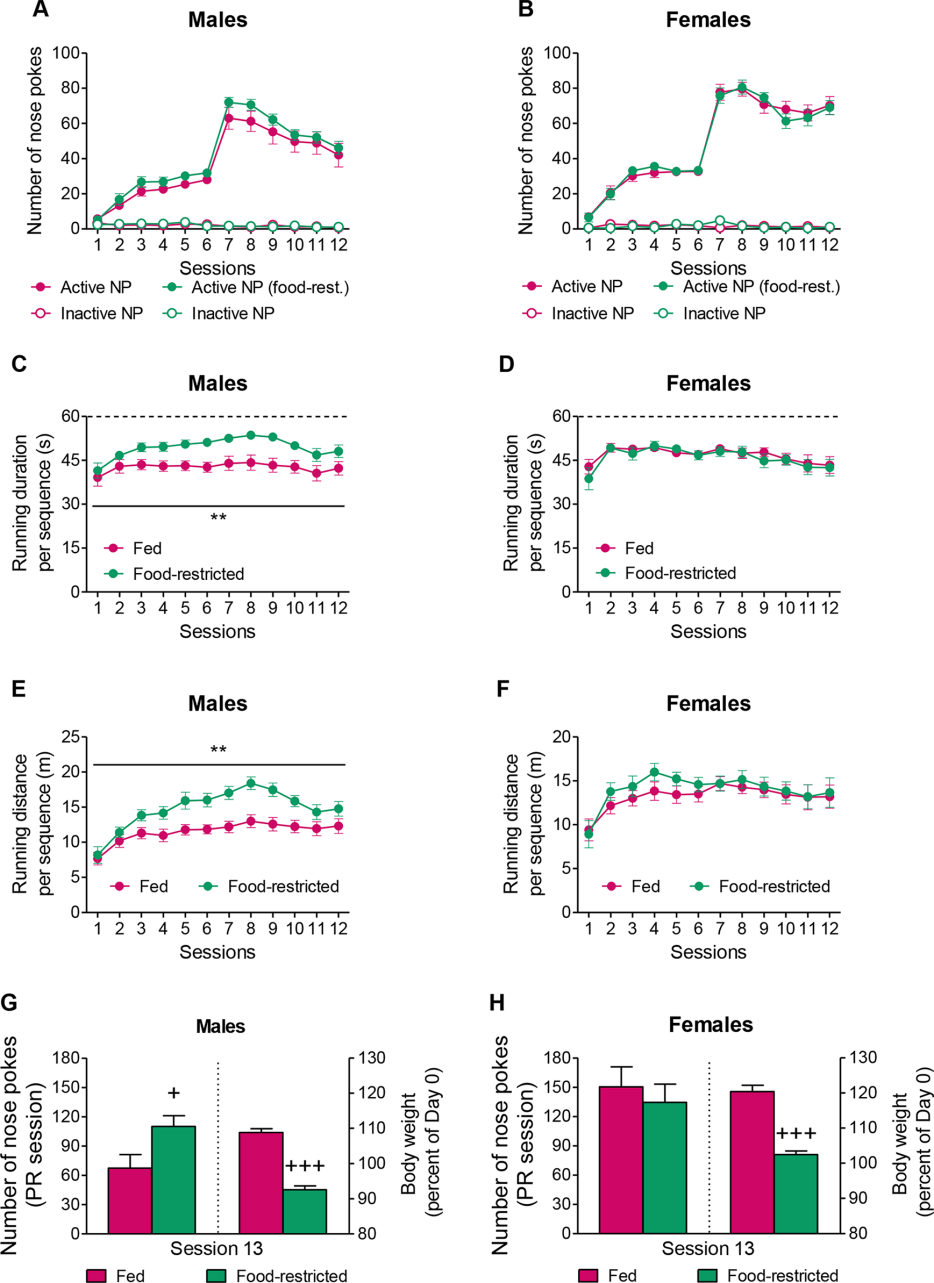
Sex-dependent effects of food restriction on running motivation and running performances. **(A,B)** Neither male mice **(A)** nor female mice **(B)** displayed changes in their nose poke responses for wheel-running with food restriction when placed under FR1/FR3 schedules of reinforcement. **(C,D)** Food restriction increased the running duration per rewarded sequence in male mice **(C)**, but not in female mice **(D)**. **(E,F)** Food restriction increased the distance ran per rewarded sequence in male mice **(E)**, but not in female mice **(F)**. **(G,H)** A food restriction regimen leading to a 15–16% reduction in body weight amplified male **(G)**, but not female **(H)** nose poke responses for wheel-running during a PR session. The values are the mean ± standard error of the mean of n = 11–14 mice. ** *p* < 0.01 for the impact of food restriction (multiple-way analysis of variance). + *p* < 0.05 and +++ *p* < 0.001 for the effect of food restriction during the PR session (Student t-test).

Taken together, the above-mentioned results indicated that PWIR amplified the stimulatory impact of food restriction on FAA in female mice. To examine whether this impact of PWIR in food-restricted mice was accounted for by specific changes in wheel-running motivation, and if so, whether these changes were sex-specific, we shifted from “free” wheel-running experiments to “effort-based” wheel-running experiments. Using operant conditioning, we first examined how food restriction affected running motivation in male and female mice before we analyzed the extent to which PWIR in male and female mice affected their motivation for i) wheel-running and ii) food intake under fed and food-restricted conditions. Food restriction did not affect male (**Figure 3A**) and female (**Figure 3B**) nose poke responses for wheel-running under FR1/FR3 schedules of reinforcement. Beside, the overall analysis of nose pokes in (fed and food-restricted) male and female mice revealed higher scores in females, as compared to males (F_1,44_ = 20.74, *p* < 0.0001; **Figure 3A and B**). As opposed to its lack of effect on nose poke responses, food-deprivation increased both the running duration per rewarded sequence (F_1,23_ = 11.82, *p* = 0.0022; **Figure 3C**) and the distance ran per rewarded sequence (F_1,23_ = 12.83, *p* = 0.0016; **Figure 3E**) in male mice, but not in female mice (**Figure 3D and F**). When tested under a PR schedule of reinforcement, fed and food-restricted females were found to perform better than their fed and food-restricted male counterparts (F_1,44_ = 10.42, *p* = 0.0024; **Figure 3G and H**), indicating higher motivation in the former mouse groups. However, when focusing on the effects of the feeding regimen on running motivation, males (**Figure 3G**), but not females (**Figure 3H**), proved sensitive to the stimulatory impact of food restriction although the latter bore sex-independent body weight-reducing effects (**Figure 3G and H**). Sex- and food restriction-dependent influences on wheel-running performances during the FR sessions extended to PR sessions as running durations per rewarded sequences (39.74 ± 2.83 s) and running distances per rewarded sequences (10.04 ± 1.22 m) were respectively increased by food restriction (47.91 ± 1.96 s and 14.81 ± 1.11 m; *p* = 0.031 and *p* = 0.011, respectively) in males, but not in females (data not shown). Taken together, these results revealed that although females displayed higher running motivation than males, their drive proved insensitive to food restriction, as opposed to that of males.

### Sex-Dependent Effects of PWIR on Nose-Poke Responding Reinforced by Wheel-Running or Palatable Food

**Figure 4:**
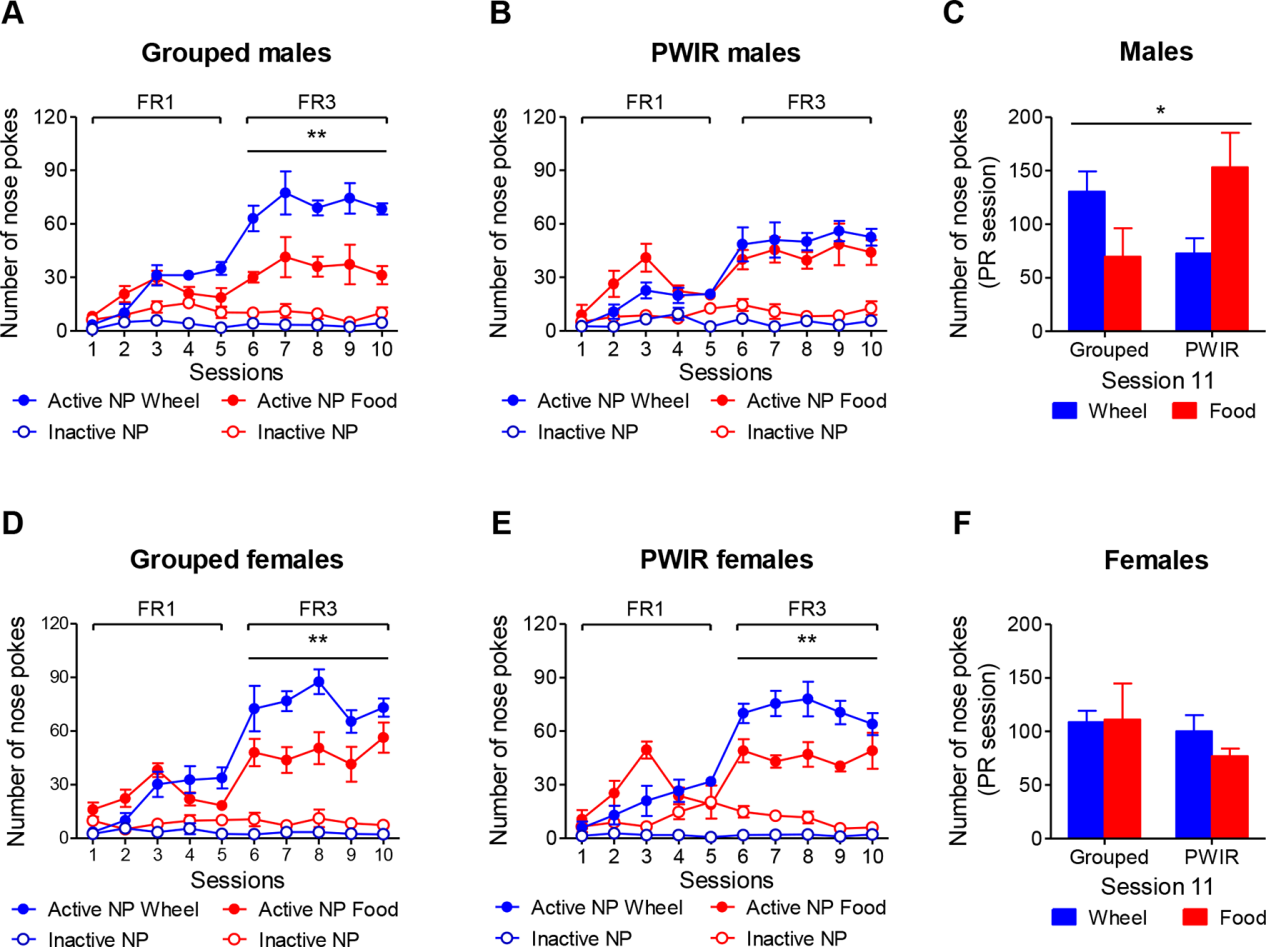
Sex-dependent effects of post-weaning isolation rearing (PWIR) on running motivation and palatable feeding motivation (each reward provided separately). **(A,B)** Grouped **(A)**, but not PWIR **(B)**, males displayed higher nose poke responses for wheel-running than for palatable food under FR3 schedules of reinforcement. **(C)** Grouped and PWIR male mice showed opposed profiles of nose poke responses for running and feeding during a PR session. **(D,E)** Both grouped **(D)** and PWIR **(E)** females displayed a higher number of nose poke responses for running, compared to feeding, under FR3 schedules of reinforcement. **(F)** Neither PWIR nor the reward nature exerted influences on the number of nose responses displayed by female mice for reward access during a PR session. The values are the mean ± standard error of the mean of n = 5–6 mice. * *p* < 0.05 for the PWIR × reward interaction during the PR session, and ** *p* < 0.01 for the overall difference between rewards under FR3 schedules of reinforcement (multiple-way analyses of variance).

The results gathered in the two preceding series of experiments rose the hypothesis that PWIR might increase nose poke responses for wheel-running in food-restricted females while possibly amplifying those evoked by food restriction in males. To test this hypothesis, we however had first to document i) the specificity of the effects of PWIR with regard to the nutritional status of the animals (*ad libitum* fed vs. food restricted), and ii) measure whether these wheel-running responses were associated with PWIR- and/or sex-dependent changes in nose poke responses for food with/without food restriction. Accordingly, we measured the respective influences of PWIR, food restriction, and sex on nose poke responses for wheel-running and palatable feeding, each reward being provided alone. Grouped (**Figure 4A**), but not PWIR (**Figure 4B**), males displayed higher nose poke responses for wheel-running than for palatable food under an FR3 schedule of reinforcement (F_1,8_ = 17.19, *p* = 0.0031). Examination of these responses under a PR schedule of reinforcement revealed a reward x housing interaction (F_1,9_ = 5.86, *p* = 0.0385) that was mainly accounted for by increased motivation for palatable food over wheel-running in isolated animals (**Figure 4C**). As opposed to males, both group-housed (F_1,8_ = 12.21, *p* = 0.008; **Figure 4D**) and PWIR (F_1,10_ = 13.49, *p* = 0.0043; **Figure 4E**) female mice responded more for wheel-running than for food under FR3 schedules of reinforcement. However, these trends did not translate into higher responses for wheel-running in the PR sessions whether nose poke numbers (**Figure 4F**) or breakpoint levels (data not shown) were considered.

### Sex-Dependent Effects of PWIR on the Choice Between Wheel-Running and Palatable Food

**Figure 5:**
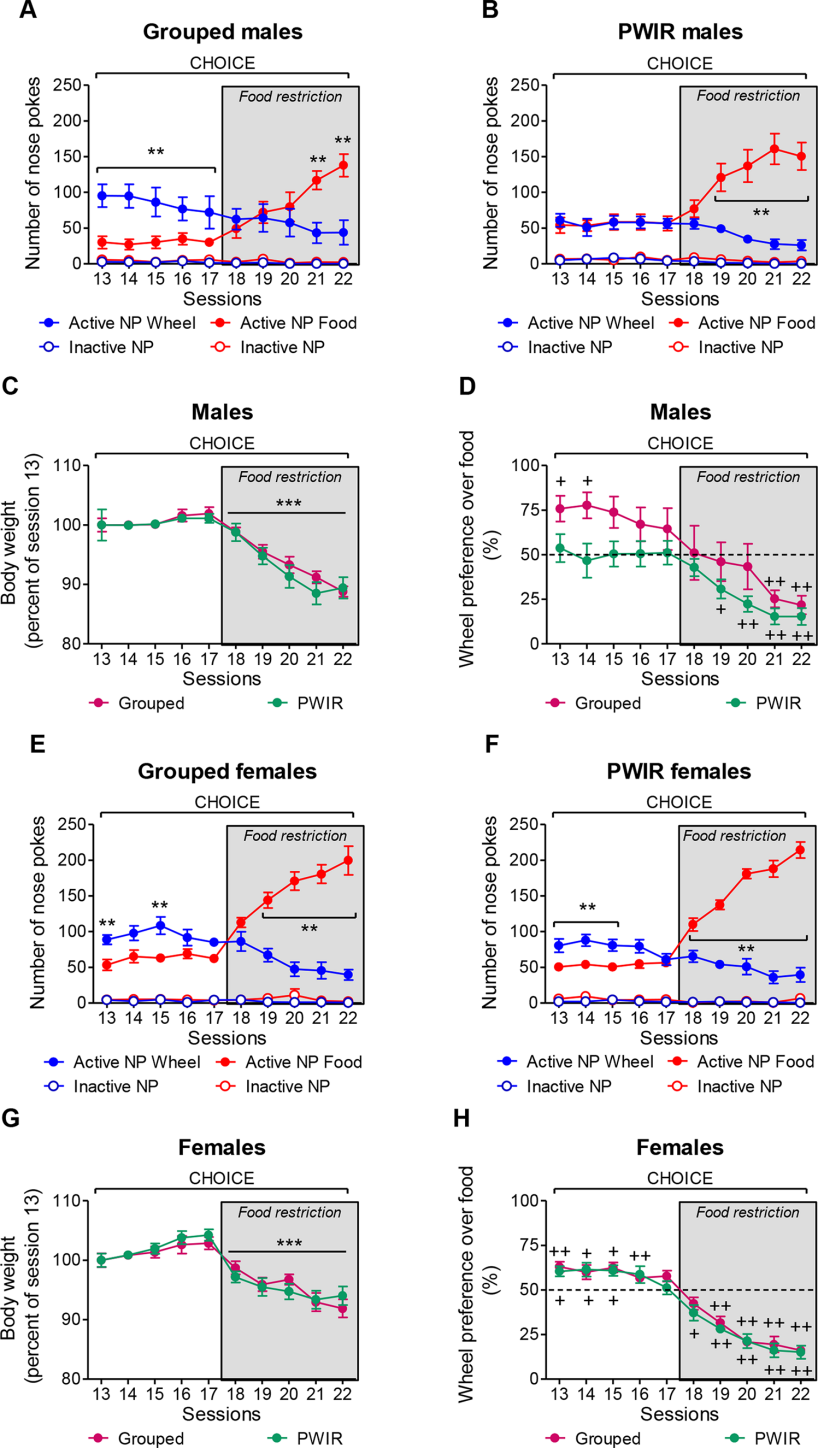
Sex-dependent effects of post-weaning isolation rearing (PWIR) on the preference between running and palatable feeding (choice sessions). **(A)** The difference in nose poke responses for running over feeding in grouped males was progressively inversed with food restriction. **(B)** Fed PWIR males displayed equal numbers of nose responses for running and feeding. **(C)** Grouped and PWIR male mice showed similar body weight losses during food restriction. **(D)** Grouped, but not PWIR, mice displayed time-dependent preferences for wheel-running over feeding. **(E,F)** The difference in nose poke responses for running over feeding in grouped and PWIR females was progressively inversed with food restriction. **(G)** Identical body weight losses in food-restricted grouped and PWIR female mice during the choice sessions. **(H)** Similar profiles of wheel-running preference over feeding in grouped and PWIR females during the choice sessions. The values are the mean ± standard error of the mean of n = 5–6 mice. ** *p* < 0.01 for the time-dependent differences between nose poke responses for wheel-running and feeding ( *post hoc* Tukey tests following significant session x reward interaction in the multiple-way analyses of variance), and *** *p* < 0.001 for the overall impacts of food restriction on body weights (multiple-way analyses of variance). ^+^ *p* < 0.05 and ^++^ *p* < 0.01 for the differences with the non-preference (50%) level (one-tailed Student t-tests).

The aforementioned experiments alternatively used wheel-running or palatable feeding as reinforcers. To examine whether the conclusions raised by these experiments extended to a reward choice situation (as daily encountered by humans, including AN patients), we performed one series of experiments wherein mice placed under an FR3 schedule of reinforcement could select one of the two rewards, this choice being temporarily exclusive. Moreover, as PWIR affected the amplitudes of the respective impacts of food restriction on body weight losses and FAA in female mice (**Figure 2B and D**), these experiments involved mice initially provided food *ad libitum* before being placed under a food restriction regimen. The analysis of the respective nose poke responses for wheel-running and palatable feeding revealed significant reward × food regimen × session interactions on nose poke responses in grouped males (F_4,32_ = 8.23, *p* = 0.0001; **Figure 5A**) and in PWIR males (F_4,40_ = 22.31, *p* < 0.0001; **Figure 5B**). However, while nose poke responses for wheel-running exceeded those for feeding during *ad libitum* feeding conditions—a difference which vanished during food restriction—in grouped males (**Figure 5A**), nose poke responses for each reward were similar in their PWIR counterparts (**Figure 5B**). Comparisons of the respective reward preference ratios in grouped males and in PWIR males (F_4,36_ = 4.90, *p* = 0.0029) confirmed these trends based on absolute nose poke responses for each reward (**Figure 5D**). Actually, the slopes of the session-dependent decreases in body weights (F_1,9_ = 67.07, *p* < 0.0001; **Figure 5C**) and wheel preference (**Figure 5D**) were similar in food-restricted grouped and PWIR males. As in males, PWIR in female mice did not affect the amplitude of body weight losses following food restriction (F_1,9_ = 54.88, *p* < 0.0001; **Figure 5G**). However, as opposed to *ad libitum* fed males, PWIR proved ineffective on the amplitude of the preference for wheel-running over feeding during *ad libitum* feeding. This was true whether absolute nose poke responses for wheel-running and palatable feeding (F_4,32_ = 20.81, *p* < 0.0001 and F_4,40_ = 19.28, *p* < 0.0001 in group-housed mice and in PWIR mice, respectively; **Figure 5E and F**) or reward preference ratios (F_4,36_ = 14.25, *p* = 0.0001; **Figure 5H**) were considered. Lastly, it is worthy of mention that the mean running preference ratio, although over 50% in grouped males (**Figure 5D**) and grouped females (**Figure 5H**), showed a sex-dependent heterogeneity of responses. Hence, in males, this heterogeneity was partly, but not fully, accounted for by one male (over five) which displayed 88–100% preference for wheel-running over feeding under *ad libitum* fed conditions before showing delayed preference for feeding, compared to the other males, under restricted conditions.

### PWIR Decreases Wheel-Running Performances in Male Mice

**Figure 6:**
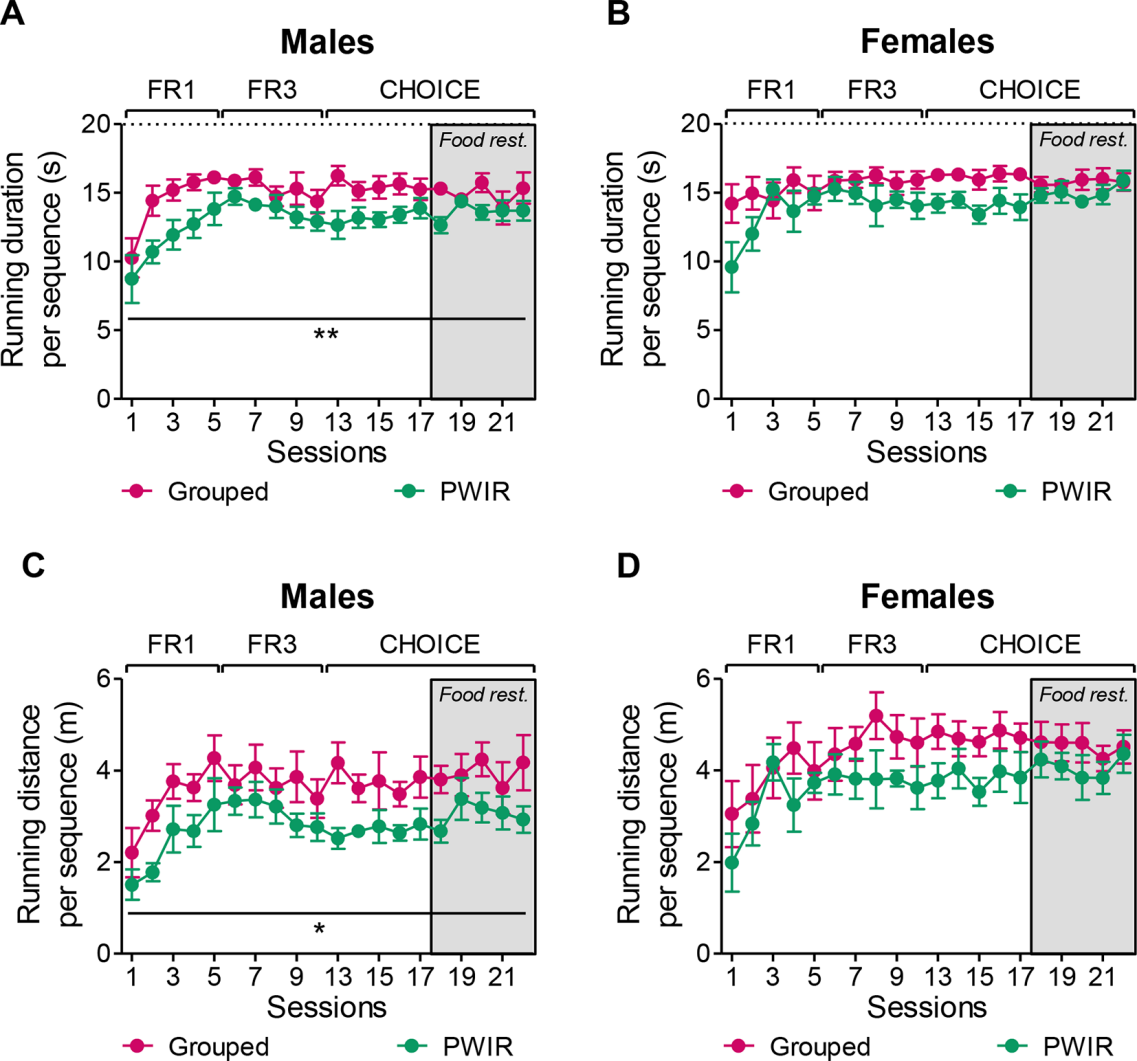
Impaired nose poke responses for wheel-running were associated with decreased running performances in PWIR males. **(A,B)** PWIR decreased the running duration per rewarded sequence in males **(A)**, but not in females **(B)**. **(C,D)** PWIR males **(C)**, but not females **(D)**, ran less distance per rewarded sequence, compared to their respective grouped controls. The values are the mean ± standard error of the mean of n = 5–6 mice. * *p* < 0.05 and ** *p* < 0.01 for the overall impacts of PWIR throughout test sessions (multiple-way analyses of variance).

The aforementioned observation that PWIR reduced the wheel preference over food in male mice might have been biased by an increased wheel-running performance during each rewarded sequence. If so, “consumption” of the reward would have compensated for decreased reward motivation in this mouse group. Analyses of wheel-running performances during each rewarded sequence argued against such a possibility. Thus, either the running duration (F_1,9_ = 10.57, *p* = 0.01; **Figure 6A**) or the running distance (F_1,9_ = 5.85, *p* = 0.039; **Figure 6C**) per rewarded sequence proved sensitive to PWIR, PWIR mice displaying decreased performances compared to group-housed mice. Indeed, these two performance indices were affected to a similar extent by PWIR, an observation which accounted for the lack of influence of that stressor on the mouse mean speed (data not shown). The impact of PWIR on wheel-running performances was sex-specific as it proved ineffective in female mice (**Figure 6B and D**).

## Discussion

AN bears the highest mortality rate among psychiatric diseases (Kaye et al., 2009), which is accounted for by our poor knowledge of its neurobiological underpinnings and hence a lack of efficient therapy for the most dramatic cases. Our ignorance of AN neurobiology lies on both its complex etiology and the translational limits of AN animal models. Although different animal models of AN exist (Mequinion et al., 2015), the one that has gained much audience is the ABA model. However, the great majority of ABA studies uses “free” wheel-running (i.e. costless access to running wheels) in their quest to elucidate the bases of AN. This can be questionned on the basis of former evidence for a motivation conflict between exercise and feeding in AN (Klein et al., 2004; Casper, 2006; Keating, 2010; Keating et al., 2012). Actually, recent observations strengthen the hypothesis of a general alteration in reward pathways in AN, whether brain responses to losses in monetary gambling tasks or therapeutic responses to the deep brain stimulation of the nucleus accumbens—a key node in brain reward pathways—are concerned (Bischoff-Grethe et al., 2013; Lipsman et al., 2017; Bernardoni et al., 2018). To date, only one study addressed the role of these pathways in the ABA model. Thus, selective chemogenetic stimulation of the dopaminergic mesoaccumbal pathway increased the percent survival to the ABA protocol, doing so by increasing food intakes and FAA-induced body weight loss in female rats (Foldi et al., 2017). An additional concern with the use of the ABA model relates to the observation that it provides neither an index of feeding motivation nor an analysis of the balance between running motivation and feeding motivation when both are available (as in the daily life of anorectics). By comparing the respective results provided by the ABA on the one hand, and reward-motivated instrumental responses on the other hand, this study provides evidence that conclusions based on the former are not valid when motivation-driven responses are considered.

As indicated above, the wide use of the ABA model is accounted for by the seminal observation that rats undergoing a food restriction regimen, i.e., a unique (time- or quantity-restricted) daily access to food, progressively increase their running performances when housed with running wheels. Actually, such an increase in performance mainly relates to FAA, a behavior classically observed in food-restricted animals prior to food presentation. The negative balance between energy intake and energy expenditure in favor of the latter thus accounts for the widespread use of ABA as an animal model of AN (although species-dependent sensitivities must be considered; Rowland et al., 2018). If so, it is expected that AN precipitating factors, such as perinatal and postnatal trauma (see Introduction), amplify such an imbalance. Actually, the use of prenatal stress, early weaning or repeated maternal separation has indicated that ABA symptomatology might be exacerbated by these procedures, albeit not necessarily in a sex-dependent manner (Glavin and Pare, 1985; Hancock and Grant, 2009; Schroeder et al., 2018). In the present study, we selected PWIR as the infant trauma. Thus, social isolation at the onset of the post-weaning period and throughout adolescence is endowed with long-lasting behavioral disturbances (e.g. anxiety, alterations in impulse control, deficit in social interactions, increased drug preference, efficient acquisition of drug self-administration; Burke et al., 2017; Walker et al., 2019) that are relevant to the scope of this study. The origins of these disturbances are likely due to the inability to express social play behavior, a highly rewarding activity that contributes to a major extent to the normal development of emotional processes (Vanderschuren et al., 2016).

As indicated above, ABA relies on a unique time- or quantity-restricted daily access to food. In most cases, a short time-window is privileged for daily food access. In our hands, preliminary observations using a daily 3-h access to food indicated that this protocol was too severe for the animals, as illustrated by precipitated and important body weight decreases that led to the discontinuation of wheel-running activity after 4 days in several animals (and hence interruption of the study for welfare reasons). Accordingly, we chose a quantity-restricted paradigm that allowed to observe significant wheel-running activity in all animals. In keeping with the aforementioned prevalence of woman suffering AN, as compared to males, we first tested whether PWIR was endowed with a significant impact in female mice exposed to an ABA paradigm. The observation that PWIR amplified the food restriction-elicited decrease in body weight—extending data in male rats (Ness et al., 1995)—while amplifying FAA, but not postprandial activity, argues against the proposal that the latter is directly related to weight loss (Wu et al., 2014). Besides putative species differences (mice vs. rats), one likely explanation for this discrepancy lies on the fact that in the latter study food-restricted animals were provided food during the light phase of the light/dark cycle (as in many other ABA studies), and not at the onset of the dark phase (present study), i.e., when rodents normally begin eating. Actually, such a time-dependent importance of food delivery, with respect to the light/dark cycle, has been documented elsewhere (Dwyer and Boakes, 1997). Thus, body weight losses, besides being of lower amplitude if food is provided at the onset of the dark period, were found to stabilize more rapidly when feeding occurred within the dark period than within the light period (Dwyer and Boakes, 1997). Noteworthy is the additional finding that the comparison between animals only allowed FAA (i.e., by unblocking the wheels during the hours preceding food provision) and animals allowed to run throughout the light/dark cycle indicated that ABA was fully accounted for by FAA (Dwyer and Boakes, 1997).

Our finding that FAA was increased in PWIR females, as compared to group-housed females, suggested that wheel-running motivation might be exacerbated in the former animals. Besides indicating the crucial need to shift to a paradigm allowing to specifically measure running motivation (Collier and Hirsch, 1971; Iversen, 1993; Belke, 1997; Muguruza et al., 2019)—doing so through the quantitation of the efforts the mice accept to provide to unlock a running wheel—this result raised two issues. The first was related to the impact of sex, if any, on running motivation in PWIR mice. The second issue involved the need to measure feeding motivation as to appreciate how PWIR might affect the balance between running and feeding drives. To explore these issues, we exposed fed/food-restricted group-housed/PWIR mice to operant protocols that specifically allow to estimate wheel-running and feeding drives as well as running performances (Muguruza et al., 2019). However, before focusing on these issues, we asked two preliminary, albeit important, questions within the present context, i.e. does food-restriction increase wheel-running motivation, and if so, is the amplitude of that increase sex-dependent? Thus, although the stimulatory impacts of either food restriction or complete fasting on wheel-running performance are known since almost 70 years (Finger, 1951), only one study, which used rats, compared males and females with respect to wheel-running motivation under fed and food-restricted conditions (Pierce et al., 1986). It was observed that the relationship between the amplitude of food restriction and running motivation, as estimated during a PR session, followed an inverted U-shaped curve with females responding to food restriction with seemingly higher running motivation than males (albeit the low number of animals impedes any conclusion; Pierce et al., 1986). The observation that food-restriction might increase running motivation fits with the finding that motivation for wheel-running under food-limited conditions is food-related, hence increasing performance, at least under “free” wheel-running conditions (Belke and Pierce, 2016). To our surprise, our female mice, albeit responding more than males for wheel-running under both constant (FR) and progressive (PR) reinforcement schedules, proved insensitive to food restriction. Conversely, food restriction stimulated male nose poke responses during the PR, but not the FR, sessions, indicating increased motivation. Interestingly, the lack of impact of food restriction on male nose poke responses during FR sessions did not extend to wheel-running performances at each rewarded sequence, as illustrated by the increased running duration/distance throughout these sessions. In keeping with our previous observation that mice bearing a deletion of the cannabinoid type-1 (CB1) receptor display decreased nose poke responses for wheel-running during FR/PR sessions without any alteration in running duration/distance at each rewarded sequence (Muguruza et al., 2019), the present study reinforces the belief that running motivation and running “consumption” (as assessed from running performances) are different entities (Belke and Garland, 2007; Muguruza et al., 2019).

That food restriction did not stimulate running motivation in our female mice although ABA-induced FAA, albeit of weak amplitude, could be observed in these animals suggested that FAA is not an index of running motivation. If so, this in turn would indicate that the aforementioned stimulatory impact of PWIR on FAA occurs without any change in running motivation. At first sight, this possibility might appear counterintuitive in keeping with the aforementioned report that chemogenetic stimulation of the mesolimbic pathway, which plays a key role in motivation for rewards, slightly, but significantly, amplifies FAA (Foldi et al., 2017). Accordingly, we analyzed wheel-running motivation in PWIR and grouped females, extending this investigation to males as running motivation was stimulated in a sex-dependent manner by food restriction. Moreover, as AN associates high exercise motivation with low feeding motivation under circumstances during which both rewards are in competition, we took advantage of our recently developed operant paradigm wherein the reinforcing values of these two rewards can be assessed separately in fed animals before being compared within a choice paradigm under fed and food-restricted conditions (Muguruza et al., 2019). Under fed conditions, whether the rewards were provided separately or within a choice paradigm, PWIR males responded to similar extents for wheel-running and for palatable food when all other mouse groups displayed increased responding for wheel-running. The negative impact of PWIR on male nose poke responding for wheel-running, compared to that measured in the other mouse groups, extended to running performance. Thus, when analyzed when wheel-running was proposed either solely or in concurrence with palatable food, the running duration/distance per rewarded sequence was decreased in PWIR males, compared to grouped males. This suggests that PWIR bears negative consequences on both wheel-running motivation and wheel-running “consumption”. Considering the finding mentioned above that wheel-running motivation is under tight control by CB1 receptors (Rasmussen and Hillman, 2011; Muguruza et al., 2019), the observation that PWIR decreases CB1 receptor activity in rats (Zamberletti et al., 2012) and mice (Muguruza et al., in preparation) might provide a route of investigation to unravel the neurobiological underpinnings of decreased running motivation in PWIR males. As concerns the reduced wheel-running performance in these animals, the finding that opioid receptors, the density of which is reduced by PWIR (Schenk et al., 1982), might control wheel-running performance without impacting on running motivation (Rasmussen and Hillman, 2011), provides another promising route of investigation. Confirmingly, opiate receptor blockade has been reported to alleviate, through decreased wheel-running, ABA severity (Boer et al., 1990). Using a food restriction protocol similar to that used in animals which were only tested for their running motivation (see above), motivation for food overpassed progressively that for running in all groups (with females reaching higher levels than males). In sharp contrast with the above-mentioned higher FAA in PWIR females, compared to grouped females, motivation for wheel-running proved insensitive to PWIR. Besides running protocol differences, the fact that another reward, namely palatable food, was accessible might explain this differential effect of PWIR. Indeed, studies from Ahmed’s group have shown that the rank of motivation for one of two rewards provided separately might be reversed when both rewards are proposed in concurrence (Cantin et al., 2010).

Taken together, the results from this study show that changes in “free” wheel-running performances, including FAA, in an ABA protocol by no means reflect alterations in the drive for running (as assessed through an effort-based protocol). Because AN imbalances in the respective drives for exercise and feeding are at the core of the pathology, our results question the translational usefulness of ABA. There are of course limits to the present study. One limit relates to the low numbers of animals which might have underpowered our analyses. Although this possibility must be taken into account, the data gathered in the present study clearly show that the measurement of FAA in the ABA protocol does not provide information on running motivation. The second limit is linked to our use of palatable food, instead of normal chow food, to assess the impact of PWIR on feeding motivation. Thus, adding food palatability to normal (i.e., chow) feeding behavior likely recruits additional central circuit components, including those projecting to the mesocorticolimbic dopaminergic system (Fulton, 2010). However, because i) only food-restricted mice do work to a significant extent to get access to normal chow food, and ii) this study wished to assess the respective impacts of PWIR on feeding drives under both *ad libitum* fed and food restriction conditions, the sole option was to use palatable food although we acknowledge the fact that such a use amplified PR nose poke responses, at least in fed animals, compared to normal chow. A third limit relates to the fact that this study involved animals tested daily for 30–60 min, hence increasing the objective value of each reward. Thus, AN patients are confronted throughout their daily life to the choice between these two rewards. A fourth limit is in keeping with former evidence for the oestrous cycle stage impacting on reward motivation (oestrus > dioestrus), at least for cocaine (Calipari et al., 2017). Although we cannot exclude that cycle variations contributed to the differential impacts of PWIR in the present study, it should be noted that its respective effects on FAA and nose poke responses under an FR3 schedule of reinforcement were studied through a successive number of days that encompassed the duration of the oestrus cycle. The fact that we did not include genetics in our study—although these are involved in AN etiology (see above)—might be considered another key limit. Hence, it might be that testing mouse lines different from the one used herein would have provided a female-specific increase in running motivation at the expense of that for feeding after PWIR. Another important limit stems from our procedure which only compared the respective drives for running and feeding under one schedule of reinforcement (i.e., FR3). Although the purpose of this study was not to compare the intrinsic rewarding values of running and feeding, a procedure which would have required different schedules of reinforcement (Hursh et al., 1988; Hursh and Silberberg, 2008), we cannot exclude that increasing the costs for each reward would have led to results differing from the present ones. As rightly proposed by Rowland et al., 2018 in their use of a cost-based anorexia model, increasing the cost to access food would mimic the high cost AN patients feel with regard to food. Accordingly, using a cost-based anorexia model wherein mice would be proposed food at progressively higher costs in their living environment (Atalayer and Rowland, 2011), and adding to that model increasing costs for running, could help to disentangle the neurobiologial grounds of AN. Such a model would prove useful for the development of pharmacological agents aimed at specifically altering the exercise/food drive balance (in either direction) for therapeutic goals.

## Ethics Statement

All protocols, which complied with the French (Décret 2013-118) and European (2010/63/EU) rules on animal experimentation, were approved by the local Ethic Committee (Comité d’Ethique 50) with agreement numbers DIR13111, 13649, 33-063-69 (F.C.) and A33-063-098 (animal facilities) provided under authority of the Préfecture de Gironde and the Ministry of Agriculture. Accordingly, the 3R-rules were followed, including through the use of the minimal number of animals per series of experiments that was required to reach conclusions. In addition, in keeping with the procedures used in this study (see the methodological outline), and which could have long-lasting consequences, all animals were only used once and sacrificed thereafter.

## Author Contributions

BR, IH, GM, and FC designed the research. BR, IH, AS, MM, and FC performed research. BR, IH, AS, MM, and FC analyzed the data. FC wrote the first version of the manuscript before it was edited and approved by all authors.

## Funding

This work was supported by Institut National de la Santé et de la Recherche Médicale (to GM), la Région Aquitaine (to GM), the University of Bordeaux (to GM), the European Research Council (ERC-2017-AdG-786467 to GM), l’Agence Nationale de la Recherche (to GM and FG), l’Agence Française de Lutte contre le Dopage (to FC), and the LabEx-Bordeaux Région Aquitaine Initiative for Neuroscience (to GM and FC).

## Conflict of Interest Statement

The authors declare that the research was conducted in the absence of any commercial or financial relationships that could be construed as a potential conflict of interest.
